# BYL719 reverses gefitinib-resistance induced by PI3K/AKT activation in non-small cell lung cancer cells

**DOI:** 10.1186/s12885-023-11243-0

**Published:** 2023-08-08

**Authors:** Yaya Yu, Zhenzhen Xiao, Chenjing Lei, Changju Ma, Lina Ding, Qing Tang, Yihan He, Yadong Chen, Xuesong Chang, Yanjuan Zhu, Haibo Zhang

**Affiliations:** 1 The Second Clinical Medical School of Guangzhou University of Chinese Medicine, The Second Affiliated Hospital of Guangzhou University of Chinese Medicine, Guangzhou, China; 2 Department of Oncology, Guangdong Provincial Hospital of Chinese Medicine, Guangzhou, China; 3 Guangdong-Hong Kong-Macau Joint Lab on Chinese Medicine and Immune Disease Research, Guangzhou, China; 4 Guangdong Provincial Key Laboratory of Clinical Research on Traditional Chinese Medicine Syndrome, Guangzhou, China; 5 State Key Laboratory of Dampness Syndrome of Chinese Medicine, The Second Affiliated Hospital of Guangzhou, University of Chinese Medicine, Guangzhou, China

**Keywords:** Non-small cell lung cancer, Epidermal growth factor receptor, PIK3CA, Phosphatidylinositol 3-kinase, Gefitinib, BYL719

## Abstract

Non-small cell lung cancer (NSCLC) patients with *epidermal growth factor receptor* (*EGFR*) mutation often obtain de novo resistance or develop secondary resistance to EGFR tyrosine kinase inhibitors (EGFR-TKIs), which restricts the clinical benefit for the patients. The activation of phosphatidylinositol 3-kinase (PI3K)/AKT signal pathway is one of the most important mechanisms for the EGFR-TKIs resistance beyond T790M mutation. There are currently no drugs simultaneously targeting EGFR and PI3K signal pathways, and combination of these two pathway inhibitors may be a possible strategy to reverse theses resistances. To test whether this combinational strategy works, we investigated the therapeutic effects and mechanisms of combining BYL719, a PI3Kα inhibitor, with gefitinib, an EGFR-TKI inhibitor in EGFR-TKIs resistance NSCLC models induced by PI3K/AKT activation. Our results demonstrated that *PIK3CA* mutated cells showed increased growth rate and less sensitive or even resistant to gefitinib, associated with increased PI3K/AKT expression. The combination of BYL719 and gefitinib resulted in synergistic effect compared with the single agents alone in *EGFR*-mutated NSCLC cells with PI3K/AKT activation. The inhibition of AKT phosphorylation by BYL719 increased the antitumor efficacy of gefitinib in these cell lines. Moreover, the combined effect and mechanism of gefitinib and BYL719 were also confirmed in the NSCLC cells and patient-derived organoids under 3D culture condition, as well as in vivo. Taken together, the data indicate that *PIK3CA* mutation induces more aggressive growth and gefitinib resistance in NSCLC cells, and the combination treatment with gefitinib and BYL719 is a promising therapeutic approach to overcoming EGFR-TKIs resistance induced by PI3K/AKT activation.

**Supplementary Information:**

The online version contains supplementary material available at 10.1186/s12885-023-11243-0.

## Introduction

With an estimated 1.8 million deaths and 2.2 million new cancer cases, lung cancer maintains the leading cause of cancer death and is the second most commonly diagnosed cancer in 2020 [1]. Non-small cell lung cancer (NSCLC) accounts for about 80–85% of all lung cancers. Epidermal growth factor receptor tyrosine kinase inhibitors (EGFR-TKIs), such as gefitinib, erlotinib, and osimertinib, have been the first line therapies for advanced NSCLC patients harboring sensitive *EGFR* mutation [2–4]. However, these patients often obtain de novo resistance or develop secondary resistance to EGFR-TKIs which restricts the clinical benefit. It has been reported that patients with abnormal activation of phosphatidylinositol 3-kinase (PI3K)/AKT pathway, which can be induced by *PIK3CA* mutation/amplification, *ErbB2* amplification, *MET* amplification, *PTEN* absence, or *AKT* amplification, usually have poorer clinical outcomes after EGFR-TKI treatment, despite of the sensitive *EGFR* mutation [5–9].In fact, about 15-48% *EGFR*-mutated NSCLC patients resistant to EGFR-TKIs harbored at least one genetic variation in PI3K/AKT pathway [8, 9]. Therefore, the abnormal PI3K/AKT pathway activation may be the most common reason for EGFR-TKIs resistance beyond the *EGFR* T790M mutation. The standard platinum-based chemotherapy for these EGFR-TKI-resistant patients may only bring a median progression free survival (PFS) time of 4 months, which was not satisfying [10, 11]. Overall, there is still lack of safe and effective treatments specific for NSCLC patients with EGFR-TKI-resistance induced by the activation of PI3K/AKT signal pathway [12].

The PI3Ks are the key proteins in the PI3K/AKT pathway, which convert PIP2 to PIP3, activating AKT to promote cell growth and proliferation. The PI3Ks can be organized into class I, class II and class III, and class IA PI3Ks play a major role in a variety of human cancers and therefore represent potential therapeutic targets [13]. Class IA PI3Ks consist of a regulatory (p85) subunit and a catalytic (p110) subunit. There are three class IA PI3K isoforms, namely, PI3Kα, PI3Kβ and PI3Kδ, with the p110 subunit encoded by *PIK3CA*, *PIK3CB* and *PIK3CD*, respectively, and the PI3Kα is the most common in solid tumors [14]. The *PIK3CA* mutation, activating PI3Kα, is one of the most common mechanisms for PI3K/AKT activation in cancers. The *ErbB2* amplification and *MET* amplification may also induce bypass activation of PI3Ks. Thus, BYL719, a selective inhibitor of PI3Kα, has been placed high expectations to inhibit PI3K/AKT activation. It has been proved to be safe and efficacious in HR (+), HER2 (-) advanced breast cancer patients harboring *PIK3CA* mutation [15, 16]. Besides, it has shown anti-cancer efficacy in squamous cell lung cancer cells with *PIK3CA* mutation in vitro and in vivo [17]. Therefore, it is worth to explore whether BYL719 may overcome EGFR-TKI resistance induced by PI3K/AKT activation. *PIK3CA* mutation, *PTEN* absence and *ErBb2* activation can activate PI3K/AKT signal pathway. Therefore, we select H1975 (*EGFR L858R* mutation and *ErBb2* activation) and H1650 (*EGFR Exon19* deletion and *PTEN* absence) to study the anti-cancer cell effects of BYL719 and gefitinib in the two NSCLC cell lines. There are no NSCLC cell lines both carrying *EGFR* mutation and *PIK3CA* mutation. Therefore, we choose the *EGFR*-mutant cell lines PC-9 and HCC-827 (gefitinib sensitive cells) to construct *EGFR*-mutant and *PIK3CA*-mutant cells by stable transfecting *PIK3CA* mutation plasmid to model the *PIK3CA* mutation-induced gefitinib insensitive cells. In the study, we detect the combined anti-cancer effects and mechanisms between the BYL719 and the gefitinib, in various of *EGFR* mutated NSCLC cells with PI3K/AKT pathway activation in vitro and *in vivo.* The models of three-dimensional (3D) spheroid and the patient-derived lung cancer organoids are also chosen to confirm these findings for the better recapitulation of in vivo morphologies [18, 19].

## Materials and methods

### Chemical reagents

Gefitinib (Selleck, China) and BYL719 (Novartis) were dissolved in dimethyl sulfoxide (DMSO) to a 20 mM concentration. DMEM/F12 medium (Cat. No.:11,330,032), B27 (Cat. No. 11,330,032), 0.25% pancreatin (Cat. No. 15,050,065) were purchased from Gibco (USA); EGF (Cat. No.: AF-100-15-100), bFGF (Cat. No. AF-100-18 C) were purchased from PeproTech Company (USA); Matrigel (Cat. No. 356,231), Cell Recovery Solution (Cat. No. 354,253) were purchased from Corning Company (U.S.); Puromycin was purchased from Mpbio Company (U.S., Cat. No. 219,453,925); Y-27,632 was purchased from AbMole (U.S., Cat. No. 129,830,382). CellTiter-Glo® 3D Cell Viability Assay was purchased from Promega (USA, Cat. No. G9682); AnnexinV-FITC/PI Apoptosis Kit was purchased from BD (USA, Cat. No. 556,547), HiScript III RT SuperMix for qPCR (+ gDNA wiper) (Cat. No. R323-01), ChamQ SYBR qPCR Master Mix (Cat. No. Q311-03) were purchased from Novozymes Biotechnology Co., Ltd. (China). The 3-(4, 5-dimethylthiazol-2-yl)-2,5-diphenyltetrazolium bromide (MTT) power was purchased from Sigma Aldrich (Mo, USA). 5-Ethynyl-2′-deoxyuridine (EdU) detection kit was ordered from Ribo Biological Co., Ltd. (Guangzhou, China). Antibodies to PIK3CA, AKT, p-AKT (Ser473), and GAPDH were purchased from Cell Signaling Technology (MA, USA).

### The construction of NSCLC cells carrying dual *EGFR* and *PIK3CA* mutations

Because there was no NSCLC cell line harboring *EGFR* and *PIK3CA* co-mutations, we used the lentiviral transfection method to stably over express *PIK3CA* mutation in PC-9 (PC-9-PIK3CA-M) and HCC-827 (HCC-827-PIK3CA-M) cells. The process for PC-9-PIK3CA-M construction and validation was the same as we previously reported [20]. The HCC-827 cells were planted into a 12-well plate with the cell density of 1 × 10^5^/well. Then the cells were transfected with 10 MOI of lentivirus containing *PIK3CA* mutation plasmid after 24 h of incubation. When the cells reached 80-90% confluence, HCC-827 cells were passaged and 1.5 µg/mL puromycin was added to the media for maintenance culture to select the HCC-827 cells overexpressing *PIK3CA* mutation plasmid (HCC-827-PIK3CA-Mutation cells, HCC-827-PIK3CA-M cells). Western blotting analysis was used to confirm the overexpression of PIK3CA and its downstream protein expression, and MTT was used to detect the gefitinib sensitivity in HCC-827 -M cells.

### 2D and 3D cell culture

Human NSCLC cell lines PC-9, HCC-827, H1650 and H1975 were obtained from the American Type Culture Collection (ATCC, Manassas, VA, USA). For NSCLC cells in 2D culture system, cells were cultured in RPMI 1640 complete medium containing 10% fetal bovine serum (FBS) at 37 °C in a humidified atmosphere containing 5%CO_2_. For the culture of HCC-827-PIK3CA-M and PC-9-PIK3CA-M cells, 1.5ug/mL and 2.5 ug/mL puromycin was add, respectively. For H1975 and H1650 cells in BME-based 3D culture system, the cells were digested and resuspended, and they were mixed evenly according to the ratio of cell suspension: Matrigel = 1:1. Matrigel solidified after incubation at 37 °C for 30 min, then 3D cell culture medium (DMEM/F-12, 20 ng/mL EGF, 10 ng/mL bFGF, 5 µM Y-27,632, 1×B27) was added based on the protocol with small modification [21]. The medium was changed every 3–4 days. For the 3D culture of PC-9-PIK3CA-M cells, additional 2.5ug/mL puromycin was add.

### Cell viability assays

MTT assay was used to determine cell viability in 2D culture. The NSCLC cells (5 × 10^3^ cells/well) in 96-well plates were cultured for 24 h at 37 °C with 5% CO2. Following treatment with cells treated with drugs alone or in combination for 24 h, 10 µL MTT solution (5 mg/ml) was added into each well and incubated with cells for 4 h at 37˚C. Then, DMSO was added to dissolve the formazan crystals. Afterwards, absorbance at 570 nm was determined using an automated microplate reader (Victor X5, Perkin Elmer, MA, USA). Cell viability (%) was calculated as follows: (OD570nm value of the experimental group/OD570nm value of the control group) ×100%. The experiment was repeated three times.

ATP method to detect the effect of drugs on cell viability in 3D culture. The NSCLC cells were plated on the bottom of the 384-well plate at a density of 5000 cells/10µL Matrigel, and cultured for 7 days. After the treatment with Gefitinib and BYL719 for 72 h, 50 µL of ATP reaction solution (DMEM/F12 and CellTiter-Glo® 3D cell viability detection reagent in a ratio of 1:1) was added to each well. The chemiluminescence value was read by a meter (BioTek, USA). Cell viability (%) = chemiluminescence value of experimental group×100%/ chemiluminescence value of control group. The experiment was repeated three times.

### Colony formation assay

Colony formation assay was performed to explore the grow speed of HCC-827 and HCC-827-PIK3CA-M cells. Briefly, 500 cells were plated into 6-well plate and were cultured for 8 days. Then cell colonies were washed with PBS, fixed with methanol, stained with 0.1% crystal violet, and captured.

### EdU assay

The NSCLC cells (5 × 10^3^ cells/well) were seeded into 96-well plates followed by treating with gefitinib/BYL719 for 24 h. Then the medium was removed and the cells was cultured in a resuspended DMEM medium with 50 µM EdU for 2 h at 37℃, stained with Apollo reaction reagent. All DNA contents of the cells were stained with DAPI. At last, an inverted fluorescence microscope (Nikon, Ts2RFL, Tokyo, Japan) was used to take pictures at × 200 magnifications. Three captured fields were selected randomly and the EdU-positive cells were calculated. The calculation formula was as follows: percentage of EdU-positive cells = (EdU-positive cells/Hoechst stain cells) × 100%. The experiment was repeated three times.

### Cell apoptosis assays

Cells in 2D culture were planted in 6-well culture plates at a density of 3 × 10^5^ cells/well. After 24 h of incubation, cells were treated with drugs alone or in combination, with the optimal concentration according to the MTT assay results. After the drug treatment for 24 h, the cells were digested with EDTA-free trypsin solution, harvested, and then resuspended in 500 µL Binding Buffer (1×) with 5 µL Annexin V-FITC and 5 µL PI. After incubation for 15 min at room temperature in the dark, the samples were analyzed using a Quanteon flow cytometer (ACEA, USA). Apoptosis rate = early apoptosis rate + late apoptosis rate. The experiment was repeated three times.

Cells in 3D culture were seeded in 24-well plates at a density of 25,000 cells/50µL Matrigel. After 7 days of incubation, drug intervention was performed. After the drug treatment for 72 h, the supernatant was discarded and Cell Recovery Solution was used to dissolve Matrigel to collect 3D cell clusters, and EDTA-free trypsin was applied to digest the cell clusters into single cell suspension. The Annexin V-FITC and PI staining assay in 3D culture was the same as described in 2D culture. The experiment was repeated three times.

### Western blot analysis

The cells in 2D culture were plated in 6-well culture plates at a density of 3 × 10^5^ cells/well. After 24 h of incubation, cells were treated with drugs alone or in combination. After 24 h treatment, the cells were lysed in lysis buffer supplemented with a protease and phosphatase inhibitors (Roche). Cells in 3D culture were seeded in 24-well plates at a density of 25,000 cells/50µL Matrigel. After 7 days of incubation, drug intervention was performed. After the drug treatment for 48 h, 3D cell clusters were harvested using Cell Recovery Solution to dissolve Matrigel, then the cells were lysed in lysis buffer supplemented with a protease and phosphatase inhibitors. The following operation was the same in cells cultured in 2D and 3D condition: The concentration of proteins was determined, and 15–20 µg protein of each group was resolved on an 10% denatured SDS-PAGE and transferred onto a polyvinylidene difluoride (PVDF) membranes (Millipore, MA, USA). After blocking nonspecific binding sites with 5% milk, the membranes were incubated with rabbit anti-human EGFR, p-EGFR, AKT and p-AKT monoclonal antibodies overnight at 4℃. Then the membranes were incubated with HRP conjugated anti-rabbit antibody for 1 h at room temperature. Finally, signals were detected using a freshly prepared ECL solution and the ChemiDoc XRS + System (Bio-Rad, Hercules, CA, USA). ImageLab software (version 4.0) was used to calculate the expression of each protein, which was normalized by GAPDH. The experiment was repeated three times.

### Determination of the antitumor effect in nude mice

The animal experiment was approved by the Animals Research Committee of Guangdong Provincial Hospital of Chinese Medicine (NO.2,022,019). Six-week-old BALB/c nude mice (18–22 g) were obtained from the Guangdong Sijiajingda Biotechnology Co., Ltd. (Guangzhou, China, License NO. SCXK (yue) 2020-0052) and kept in the Animal Center of Guangdong Provincial Hospital of Chinese Medicine (License NO. SYXK (yue) 2018-0094). Nude mice were injected subcutaneously with PC-9-PIK3CA-M cells (2 × 10^6^). When tumor sizes reached 100–150 mm^3^, mice were randomized into four groups of 6–8 mice each. Each group of mice was dosed via daily oral gavage with vehicle, gefitinib (2.5 mg/kg/d), BYL719 (5 mg/kg/d), or a combination of both. The gefitinib and BYL719 contained 0.5% hydroxypropyl methylcellulose. Tumor volumes were determined using calipers and were calculated using the following formula: V= (L × W^2^)/2 (L, Length; W, width). Toxicity was monitored according to weight loss. The tumor size and mice weight were detected every 3–4 day. After the treatment for total 21 days, the tumors were removed for Western blot analysis and immunohistochemistry (IHC).

### IHC

For IHC staining, the sections in paraformaldehyde were applied to block endogenous peroxide activity, and then were boiled in 0.01 M citrate buffer (pH 6.0) twice with an autoclave. Tissue sections were incubated with rabbit anti-human PIK3CA (1:100), AKT (1:200), p-AKT (1:50) monoclonal antibodies at 4˚C overnight after blocking. The sections were incubated with HRP conjugated anti-rabbit antibody after washing, and the peroxidase reaction was developed with diaminobenzidine substrate kit. Hematoxylin was then used for nucleus staining.

### The establishment of patient-derived organoid of NSCLC

Malignant pleural effusion (PE) samples were obtained from Guangdong Provincial Hospital of Chinese Medicine with consent from patients diagnosed with lung adenocarcinoma. The human experiment was approved by the Humans Research Committee of Guangdong Provincial Hospital of Chinese Medicine (NO.BF2020-017.2). Inclusion Criteria: patients with non-small cell lung cancer; patients had a genetic diagnosis of *EGFR* mutation combined with *PIK3CA* mutation. Exclusion criteria: patients over 85 years of age; patients with severe heart disease. Samples were transferred into 50 mL centrifuge tubes and centrifuged to separate cells from fluid and plasma [22]. Once cells were pelleted, BD PharmLyse was used to lyse and subsequently remove red blood cells (BD Biosciences, CA) [22]. Then cells were harvested after centrifugation, and were washed twice with advanced DMEM/F12. The strained cells were centrifuged at 200 × g for 5 min, and the pellets were resuspended in 50 µL Matrigel and later were added advanced DMEM/F12 supplemented with 20 ng/ mL of bFGF, 50 ng/mL human EGF, N2, B27, 10 µM ROCK inhibitor and 1% penicillin/streptomycin [19].

### Statistical analysis

All the methods above were performed in accordance with relevant guidelines and regulations. All numerical data are presented as the mean ± standard deviation (x ± SD). Statistical analyses were carried out using GraphPad Prism (GraphPad software). Statistical analysis between multiple comparisons was performed by one-way analysis of variance, and the significance of differences between two groups was analyzed using t tests. A p value of < 0.05 was considered as statistical significance. We applied the combination index (CI) to evaluate the drug combination effect. Combination index (CI) data were generated using CompuSyn (Combosyn). A CI of 1 indicated an additive drug interaction, whereas a CI of < 1 was synergistic and a CI of > 1 was antagonistic.

## Results

### *PIK3CA* mutation leads to faster grow and less gefitinib sensitivity in NSCLC cells

#### *PIK3CA* mutation leads to faster grow and less gefitinib sensitivity in HCC-827-PIK3CA-M cells

**Fig. 1:**
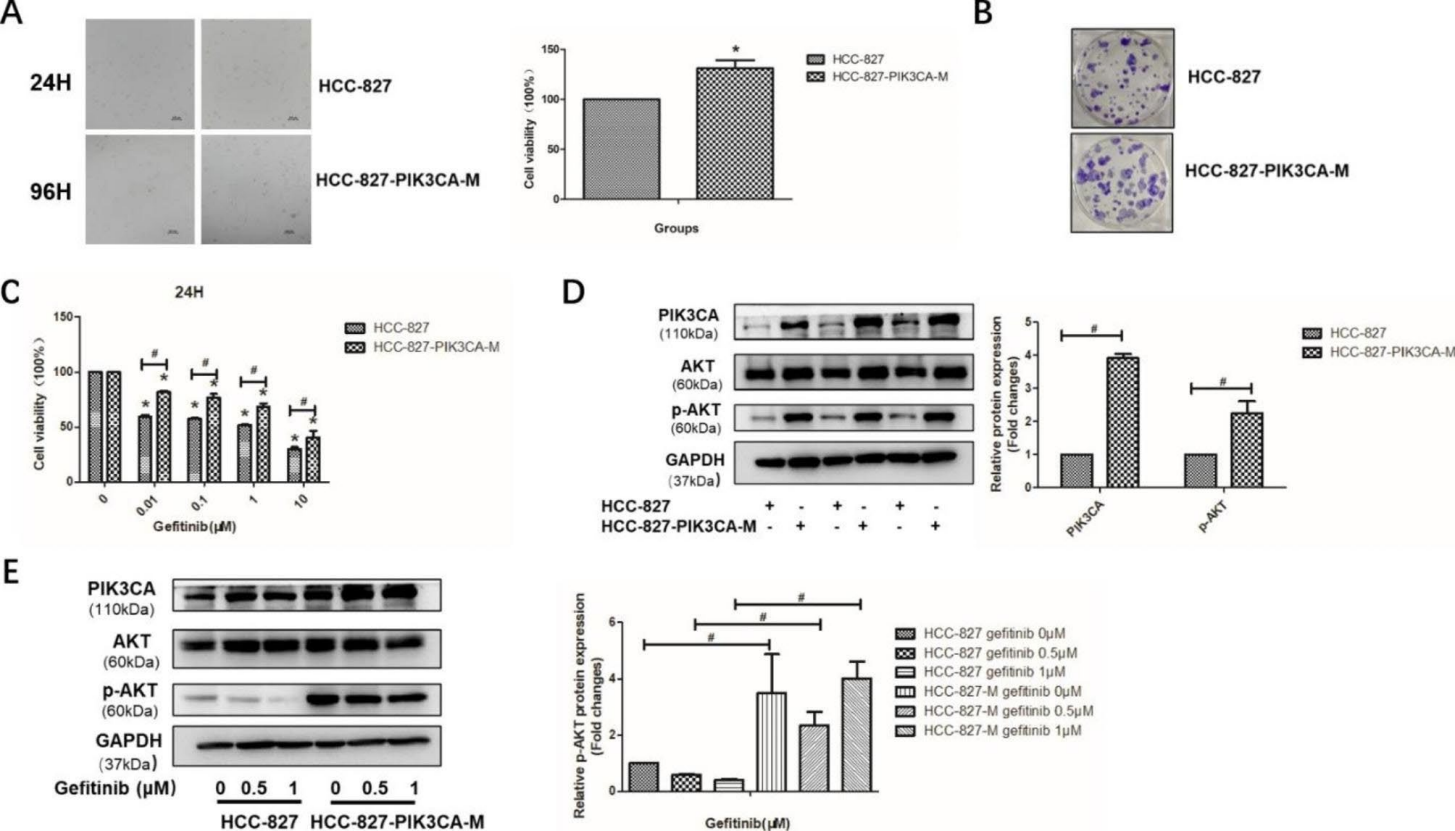
HCC-827-PIK3CA-M Cells grew faster and were less sensitive to gefitinib due to upregulated phosphorylation of AKT protein **A.** The Cell morphology and cell viability of HCC-827 and HCC-827-PIK3CA-M cells; **B.** The colony formation of HCC-827 and HCC-827-PIK3CA-M cells; **C.** The expression of PIK3CA and p-AKT protein in HCC-827 and HCC-827-PIK3CA-M cells; **D**. The cell viability detected using MTT assay after gefitinib treatment in HCC-827 cells and HCC-827-PIK3CA-M cells; **E**. The expression of PIK3CA and p-AKT protein after the treatment of gefitinib in HCC-827 cells and HCC-827-PIK3CA-M cells. *P < 0.05, Gefitinib group vs. Control group; ^#^ P < 0.05, HCC-827-PIK3CA-M group vs. HCC-827-PIK3CA-M group

HCC-827 cells were transfected with lentivirus stably over expressing *PIK3CA* mutation to construct HCC-827-PIK3CA-Mutation (HCC-827-PIK3CA-M) cells. First of all, we seeded the same number of HCC-827 and HCC-827-PIK3CA-M cells in 96-well plates and 6-well plates, and we applied the MTT assay and colony formation assay to detect the cell proliferation of the two cell lines. As shown in Fig. 1A-B, HCC-827-PIK3CA-M cells grew faster than HCC-827 cells (P < 0.05). The protein expression levels of PIK3CA and p-AKT was up-regulated in HCC-827-PIK3CA-M cells (Fig. 1C, P < 0.05). MTT results indicated that HCC-827-PIK3CA-M cells were less sensitive to gefitinib, when compared with HCC-827 cells (Fig. 1D, P < 0.05). The expression of p-AKT was significantly higher in HCC-827-PIK3CA-M cells than that in HCC-827 cells under the same dose of gefitinib, respectively (Fig. 1E, P < 0.05). These results together indicated that HCC-827-PIK3CA-M grew faster and were less sensitive to gefitinib than the parental cells due to upregulated phosphorylation of AKT protein.

#### *PIK3CA* mutation leads to over-growth and gefitinib resistance in PC-9-PIK3CA-M cells

**Fig. 2:**
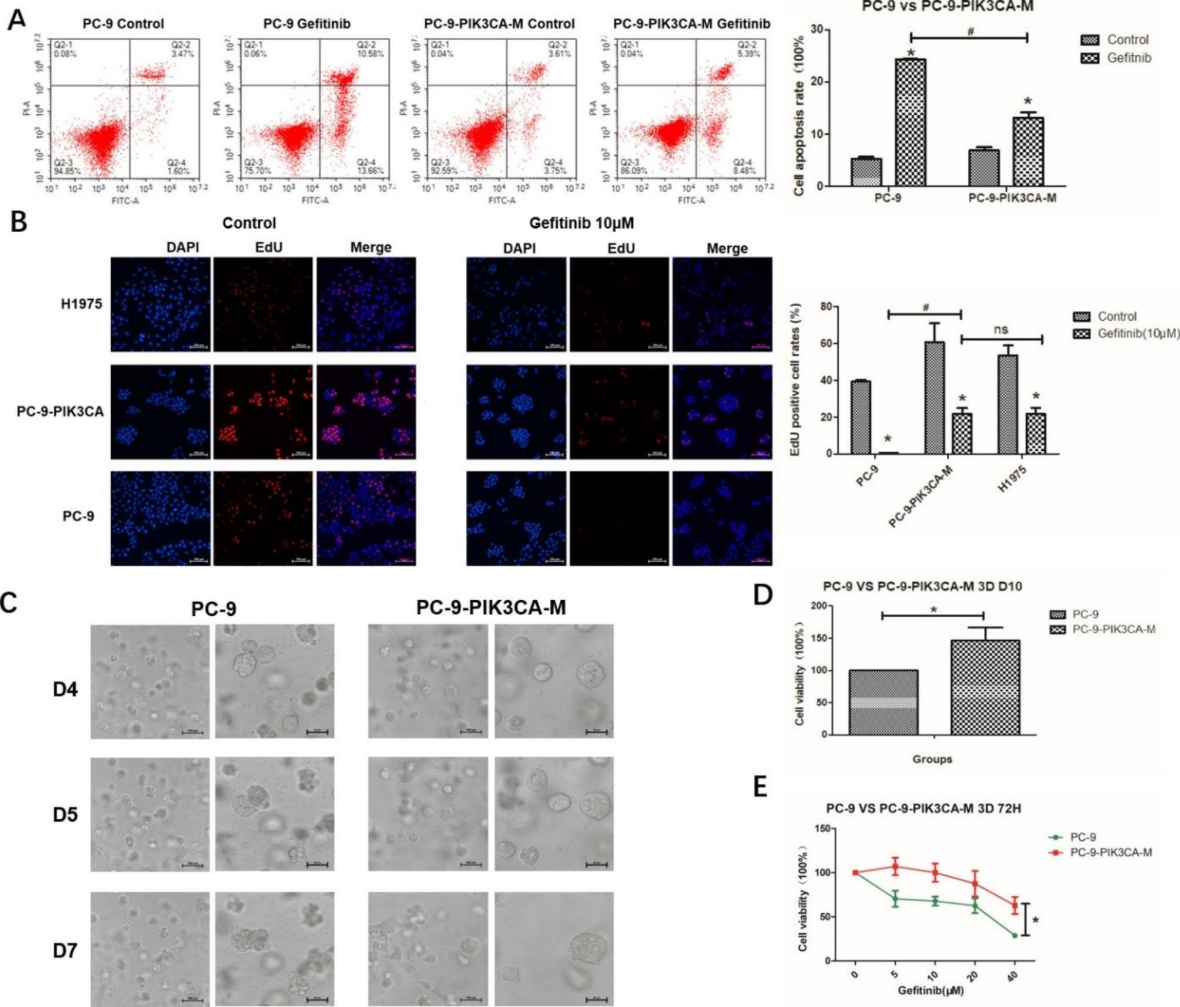
The PC-9-PIK3CA-M Cells were resistant to gefitinib. **A**. The EdU (+) rates after the treatment of gefitinib in PC-9, PC-9-PIK3CA-M and H1975 cells. **B.** The morphology of PC-9 cells and PC-9-PIK3CA-M cells in 3D culture with Matrigel on day 4, 5, 7; **C**. The cell viability (ATP method) of PC-9 and PC-9-PIK3CA-M cells in 3D culture with Matrigel on day 10; **D**. The cell viability (ATP method) after gefitinib treatment in PC-9 and PC-9-PIK3CA-M cells in 3D culture with matrigel. *P < 0.05, Gefitinib group vs. Control group; ^#^P < 0.05, PC-9-PIK3CA-M group vs. PC-9 group

We have demonstrated that gefitinib showed weaker anti-cell viability in PC-9-PIK3CA-Mutaiton (PC-9-PIK3CA-M) cells than in PC-9 cells, due to upregulated p-AKT [20]. In the present study, the significantly reduced pro-apoptotic and anti-proliferation effect of gefitinib in PC-9-PIK3CA-M cells were further confirmed, compared to PC-9 cells (Fig. 2A-B, P < 0.05). In addition, the results of EdU assay indicated that the PC-9-PIK3CA-M cells were as resistant to gefitinib as H1975 cells, a widely used gefitinib-resistant cells (Fig. 2B).

We further compared the growth rate and gefitinib sensitivity of PC-9-PIK3CA-M to PC-9 in 3D models cultured in Matrigel. The spheroid size and cell viability of PC-9-PIK3CA-M-3D cells were significantly higher than those of PC-9-3D cells (Fig. 2C-D). Consistent with the results of 2D culture, PC-9-PIK3CA-M-3D cells were more resistant to gefitinib than PC-9-3D cells (Fig. 2E, P < 0.05). These results together indicated that *PIK3CA* mutation may lead to over growth and gefitinib resistance in PC-9-PIK3CA-M cells.

### Gefitinib and BYL719 exhibited synergistic effects in *EGFR* mutated NSCLC cells with PI3K/AKT pathway activation

#### Gefitinib and BYL719 exhibit synergistic effects

In addition to the *PIK3CA/EGFR* co-mutated PC-9-PIK3CA-M and HCC-827-PIK3CA-M cells, we also used H1975 and H1650 lung cancer cells to evaluate the synergetic effects of gefitinib and BYL719. The H1975 cells harbored *EGFR* L858R mutation with *ErBb2* amplification (Supplementary Table 1) [23, 24]. The H1650 cells were with *EGFR* Exon19 deletion and *PTEN* deletion (Supplementary Table 1) [24]. The over-expression of PIK3CA was also confirmed in H1975 and H1650 cells (supplementary Fig. 1).

**Fig. 3:**
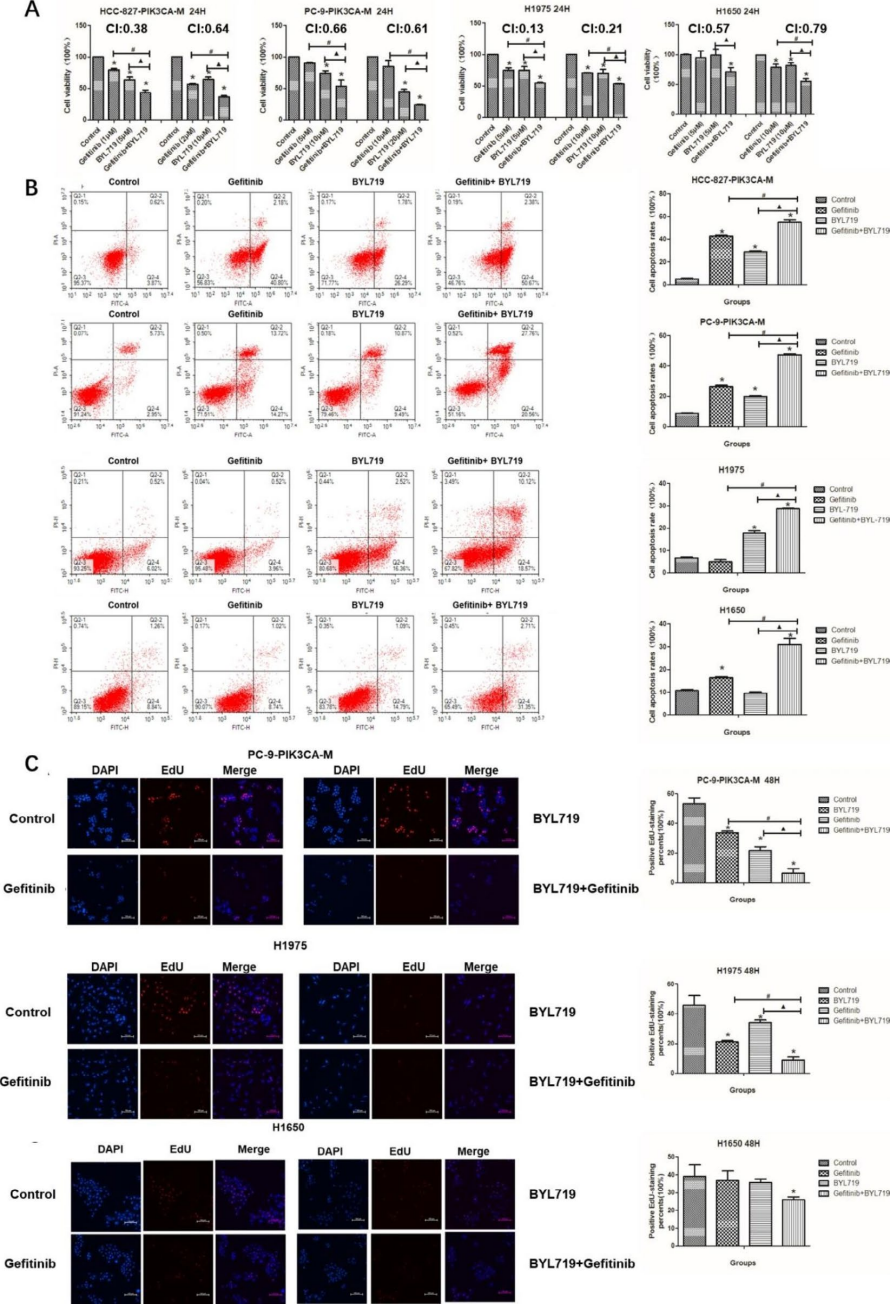
The effects of gefitinib and BYL719 on NSCLC cells. **A**: The cell viability after the treatment of gefitinib and BYL719 in HCC-827-PIK3CA-M cells, PC-9-PIK3CA-M cells, H1975 cells and H1650 cells; **B**: The cell apoptosis rates after the treatment of gefitinib and BYL719 in HCC-827-PIK3CA-M cells, PC-9-PIK3CA-M cells, H1975 cells and H1650 cells; **C**: The EdU (+) cell rates after the treatment of gefitinib and BYL719 in PC-9-PIK3CA-M cells, H1975 cells and H1650 cells. *P < 0.05, experiment group vs. Control group; ^#^P < 0.05, Gefitinib + BYL719 group vs. Gefitinib group; ▲P < 0.05, Gefitinib + BYL719 group vs. BYL719 group

MTT assay indicated that combination of gefitinib and BYL719 inhibited cell viability significantly, compared with gefitinib alone or BYL719 alone, in all of the four cell lines with *EGFR* mutation and PI3K/AKT pathway activation (Fig. 3A). All of the combination indexes (CIs) for gefitinib and BYL719 in the four cell lines were less than 1, indicating a synergistic effect (Fig. 3A).

The apoptosis rates of cells in combination group were also significantly higher than those in gefitinib group or BYL719 group, in all of the four cells (Fig. 3B). EdU assay showed that proliferation rates of cells in combination group were also significantly lower than those in gefitinib group or BYL719 group in the PC-9-PIK3CA-M and H1975 cells (Fig. 3C, P < 0.05). In the H1650 cells, gefitinib alone or BYL719 alone did not inhibit the cell proliferation, while the combination of the two drugs significantly reduced the cell proliferation, compared with the control group (Fig. 3C, P < 0.05).

#### Gefitinib and BYL719 exhibit synergistic effects by inhibiting p-AKT

**Fig. 4:**
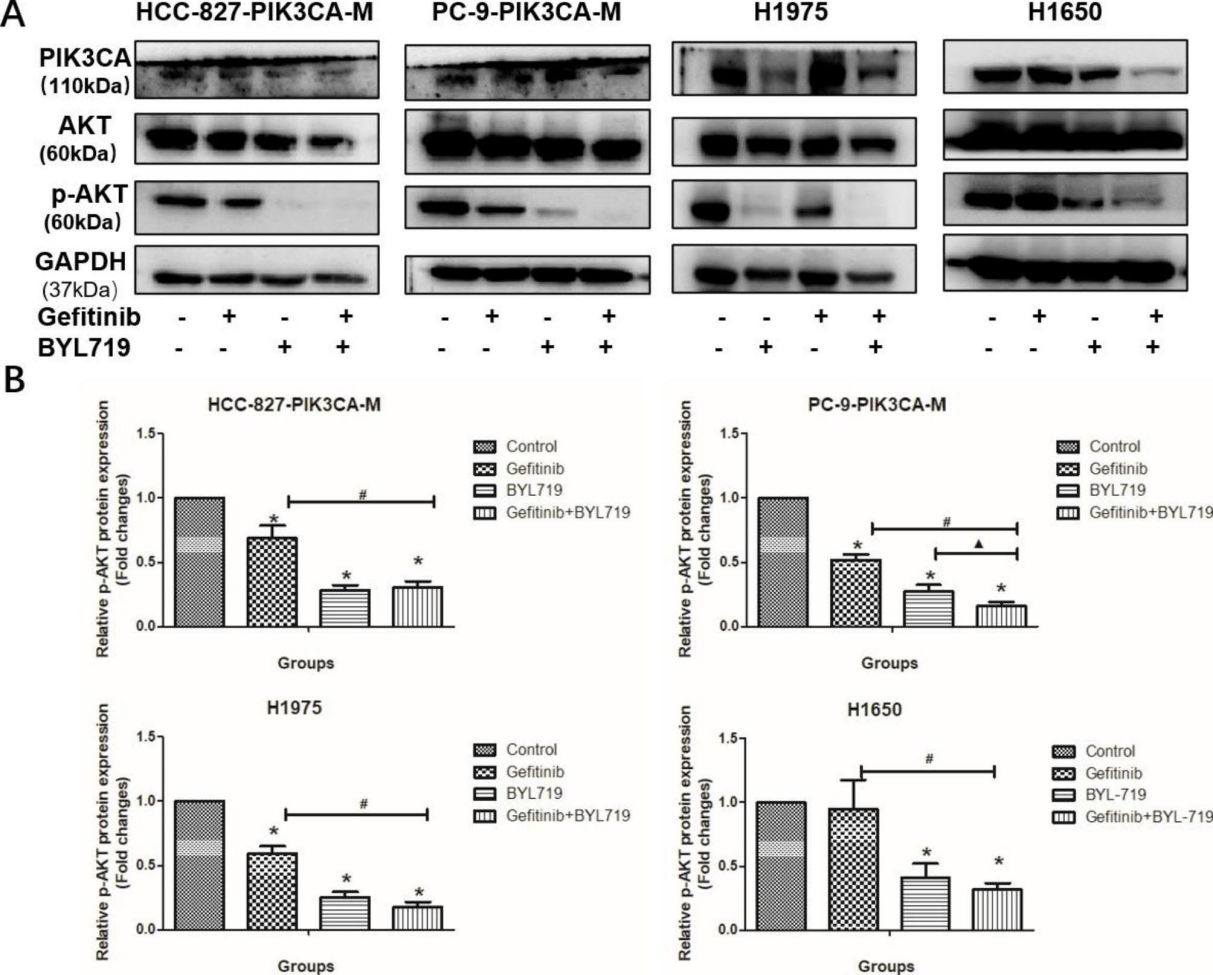
The effects of gefitinib and BYL719 on PI3K/AKT signal pathway. **A**-**B**: The protein expression of PIK3CA and p-AKT after the treatment of gefitinib and BYL719 in HCC-827-PIK3CA-M, PC-9-PIK3CA-M, H1975 and H1650 cells (A: band graphics; B: statistical graphics). *P < 0.05, Experiment group vs. Control group; ^#^P < 0.05, Gefitinib + BYL719 group vs. Gefitinib group; ▲P < 0.05, Gefitinib + BYL719 group vs. BYL719 group

HCC-827-PIK3CA-M, PC-9-PIK3CA-M, H1975 and H1650 cells were treated with BYL719 and Gefitinib, and expression levels of PIK3CA, p-AKT were measured at 24 h. The BYL719 and the combined groups showed much stronger anti-p-AKT ability in the four cell lines (Fig. 4, P < 0.05). The results suggested that BYL719 enhanced the anti-cancer effect of gefitinib by inhibiting the p-AKT signal pathway in PI3K/AKT activation-induced gefitinib-resistance NSCLC cells.

#### Gefitinib and BYL719 exhibited synergistic effects in 3D culturing models and lung adenocarcinoma organoids

**Fig. 5:**
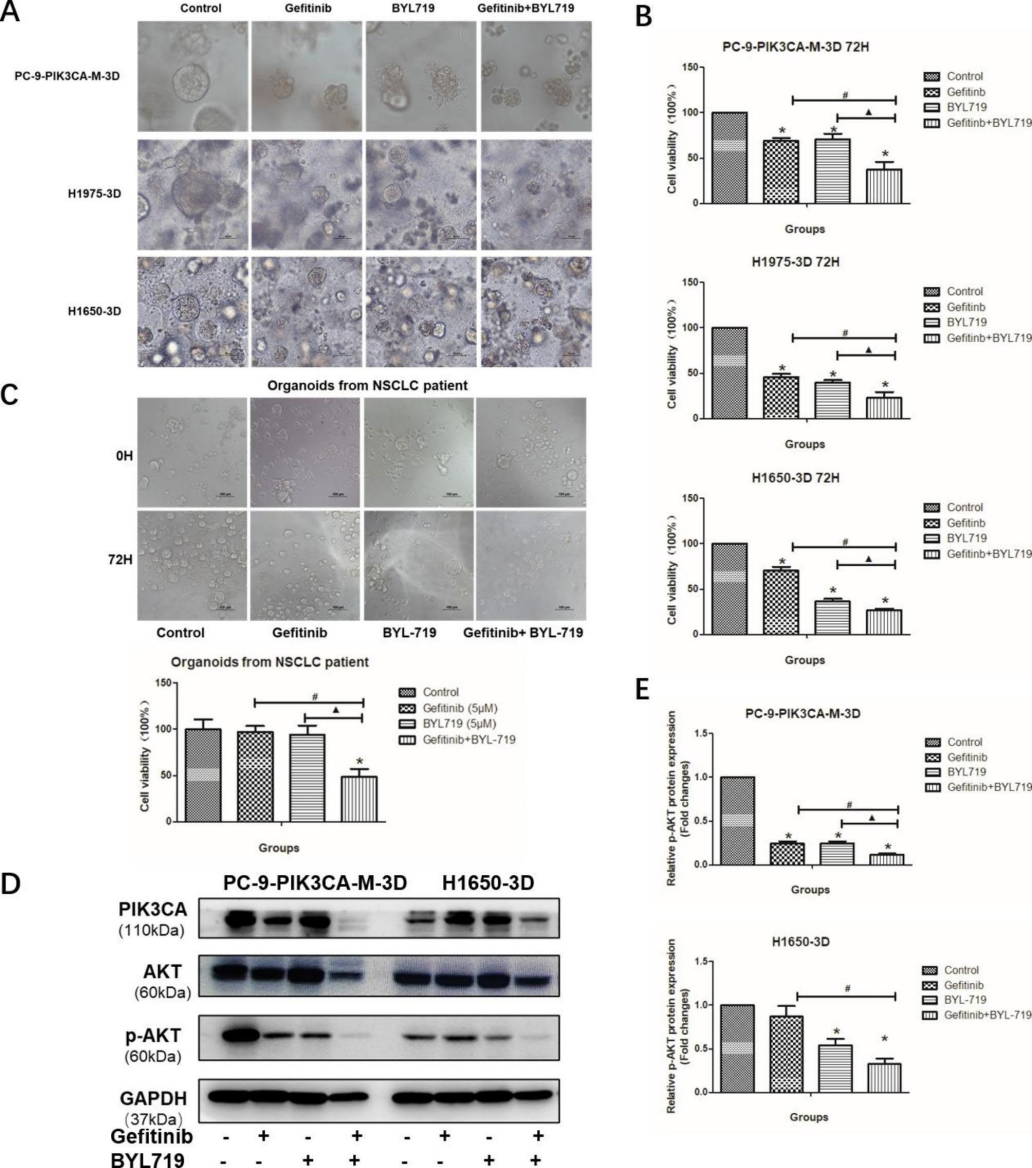
The combination effect of gefitinib and BYL719 in 3D culture. **A**-**B**: The morphology (A) and cell viability (B) after the treatment of gefitinib and BYL719 in PC-9-PIK3CA-M, H1975 and H1650 cells in 3D culture; **C**: The morphology of the patient-derived organoid of NSCLC after the treatment of gefitinib and BYL719; **D**-**E**: The protein expression of p-AKT after the treatment of gefitinib and BYL719 in PC-9-PIK3CA-M and H1650 cells in 3D culture (D: band graphics; E: statistical graphics). *P < 0.05, experiment group vs. Control group; ^#^P < 0.05, Gefitinib + BYL719 group vs. Gefitinib group; ▲P < 0.05, Gefitinib + BYL719 group vs. BYL719 group

The sizes of the cell mass became smaller, and the structure of the cell mass was destroyed after the treatment of gefitinib or BYL719, which was most obvious in the combined group (Fig. 5A). The ATP assay detecting the cell viability further confirmed that the combined group showed the strongest anti-cancer cell viability of the PC-9-PIK3CA-M, H1975 and H1650 cells among the four groups (Fig. 5B, P < 0.05). In addition, we detected the synergistic effect of gefitinib and BYL719 in organoids derived from a lung adenocarcinoma patient, harboring *EGFR* (L838V, L861Q), *PIK3CA* (H1047L), *ErbB2* (D871N) mutations and *MET* amplification. The morphology changes and the cell viability of the organoids from lung adenocarcinoma patient were shown in Fig. 5C. The results showed that both the gefitinib group and the BYL719 group had no significant effects on the organoid sizes and cell viability, when compared with the control group. While the combination of gefitinib and BYL719 significantly inhibited the growth of the organoids, showing a proliferation inhibition rate of about 60% (Fig. 5C, P < 0.05). Taken together, the combination of gefitinib and BYL719 exhibited stronger anti-cancer effects than gefitinib or BYL719 alone.

Consistent with the results of 2D culture, the p-AKT expression of the combinational group was much lower than that of control group and gefitinib group in the PC-9-PIK3CA-M and H1650 cells (Fig. 5D-E, P < 0.05). Again, these results suggested that BYL719 enhanced the anti-cancer effect of gefitinib by inhibiting p-AKT in the PI3K/AKT activation-induced gefitinib-resistance NSCLC cells in 3D culture.

#### The combination of gefitinib and BYL719 leads to enhanced anti-tumor efficacy by inhibiting p-AKT in the PC-9-PIK3CA-M xenografts

**Fig. 6:**
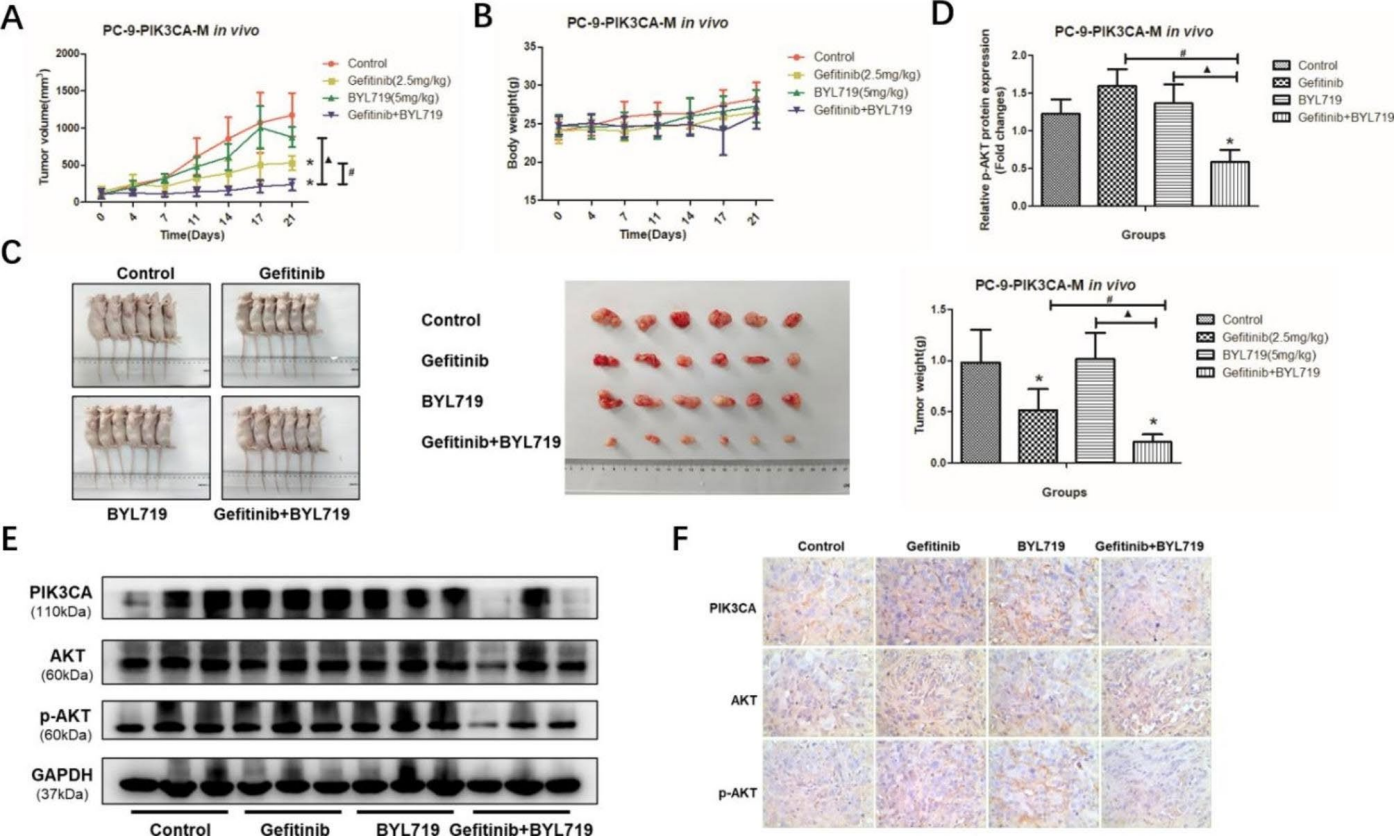
The combination effect of gefitinib and BYL719 *in vivo.***A**-**B**: The tumor volume (A) and mice weight (B) after the treatments of gefitinib and BYL719 in PC-9-PIK3CA-M xenografts for 21 days; **C**: The tumor size and the tumor weight after the treatments of gefitinib and BYL719 at 21 days in PC-9-PIK3CA-M xenografts; **D**-**F**: The protein expression of p-AKT after the treatments of gefitinib and BYL719 in PC-9-PIK3CA-M xenografts (D: statistical graphics; E: western blot; F: IHC). *P < 0.05, experiment group vs. Control group; ^#^P < 0.05, Gefitinib + BYL719 group vs. Gefitinib group; ▲P < 0.05, Gefitinib + BYL719 group vs. BYL719 group

In light of the synergistic effects of the combination therapy observed in vitro, we investigated the efficacy of combining gefitinib and BYL719 in vivo. Mice bearing PC-9-PIK3CA-M xenografts were treated with vehicle, gefitinib, BYL719, or a combination of gefitinib and BYL719. As shown in Fig. 6A, gefitinib alone and combination of gefitinib and BYL719 could significantly inhibit the tumor growth, when compared with the vehicle (P < 0.05). Moreover, the combination of gefitinib and BYL719 could significantly inhibit the tumor growth, when compared with the gefitinib group or BYL719 group (P < 0.05, Fig. 6A). The size and weight of tumor at Day 21 were shown in Fig. 6C, and the effects of drugs on tumor weigh were the same as the effects of drugs tumor volume (P < 0.05). Furthermore, no significant difference in body weight were found between the four groups during the 21 days of treatment, and no obvious toxicities were observed (P < 0.05, Fig. 6B). To investigate the pharmacodynamic effects of combining gefitinib and BYL719 in vivo, tumor lysates of PC-9-PIK3CA-M xenografts were collected at the final day, and were analyzed for PI3K/AKT expression by western blot and IHC. Consistent with the in vitro findings above, combination therapy inhibited p-AKT (P < 0.05, Fig. 6D-F). Taken together, these results suggest that the combination of gefitinib and BYL719 has a synergistic therapeutic effect on PI3K activation NSCLC by inhibiting phosphorylation of AKT in vivo.

## Discussion

The use of EGFR-TKIs for the treatment of NSCLC patients with sensitive *EGFR* mutation has created a precedent for molecularly targeted therapy. However, the problem of drug resistance largely limits the clinical benefits of EGFR-TKIs. *PIK3CA* mutations or gains are present in a subset of NSCLC with *EGFR* mutation and are associated with malignant biological behavior and EGFR-TKIs resistance in lung cancer [5–7, 9]. Analysis of a 1122 *EGFR*-mutant patient cohort revealed that multiple co-occurring oncogenic events are present in most advanced-stage EGFR-mutant lung cancers, including *PIK3CA* mutation [6]. Our previous clinic data also suggested that the *PIK3CA* co-variation, accounting for 16.67% of *EGFR*-mutant lung cancer patients, was associated with poorer PFS, only 4.57 months, after EGFR-TKI treatment [9]. *PIK3CA* alterations upregulate PI3K activity and p-AKT expression, which then promotes cell growth and proliferation [15]. In squamous cell lung cancer cells, it has been observed that newly generated *PIK3CA-*mutated SQCLC cells showed increased growth rate and enhanced migration and invasiveness[17]. Knockdown of *PIK3CA* inhibited colony formation of lung cancer cell lines with *PIK3CA* mutations or gains but was not effective in *PIK3CA* wild-type cells [5]. Our data also indicated that *PIK3CA* mutation may lead to over-growth in *EGFR* mutated NSCLC cell lines. In addition, we demonstrated that the gain of *PIK3CA* mutation may lead to less sensitivity and even resistance to gefitinib in *EGFR* mutation NSCLC cell lines, due to its activation of PI3K/AKT signal pathway. Therefore, the poor clinical outcomes of patients with EGFR/PIK3CA co-mutation resulted not only from the upregulated growth and aggressiveness, but also from the EGFR-TKI resistance due to PI3K/AKT activation.

For patients with *EGFR* combined with other mutations, the PFS after EGFR-TKIs treatment was significantly lower than that of patients with single *EGFR* mutation [25]. As mentioned before, canonical *EGFR* driver mutations co-occur with *PIK3CA* mutation [6]. *PIK3CA* and *ErbB2* gene co-variation most associated with primary EGFR-TKIs resistance in *EGFR*-mutant lung cancer patients [9]. *ErbB2* amplification, *PTEN* deletion and *PIK3CA* mutation were the common secondary EGFR-TKIs resistance mechanisms other than T790M mutation [7]. *PIK3CA* mutation activates the PI3K/AKT pathway [5, 20]. *ErbB2* amplification activate the downstream PI3K/AKT signal pathway, and the PTEN/PI3K/AKT pathway regulates multiple cellular functions, including cell growth, differentiation, proliferation, invasion and intracellular trafficking in NSCLC [26, 27]. 14.9% patients resistant to EGFR-TKIs had at least one genetic variation in PI3K pathway [8]. Based on the clinic data, the activation of PI3K/AKT pathway is an important reason for both the primary and secondary drug resistance of EGFR-TKIs for NSCLC patients. Platinum based chemotherapy was recommended as second-line treatment for patients with EGFR TKIs-resistance based on the NCCN guideline. For *EGFR* mutant patients who received platinum-based chemotherapy after disease progression with first-line EGFR TKI treatment, the response rates were only 14–18%, which is far away from our expectation [10, 11]. There is still lack of effective treatments specific for *EGFR* mutant patients with EGFR-TKI-resistance induced by the activation of PI3K/AKT signal pathway [12].

A potential strategy to overcome this resistance is to combine the EGFR-TKIs with a PI3K/AKT pathway inhibitor, including PI3K inhibitors, AKT inhibitors and mTOR inhibitors [14]. In 2009, researchers have tried this strategy, using everolimus, a mTOR inhibitor, and found that everolimus plus gefitinib induced a growth-inhibitory effect in gefitinib-resistant NSCLC cell lines[28]. However, this combination failed in clinic, with limited antitumor activity in EGFR mutant NSCLC patients with PI3K pathway aberrations[8]. In fact, the PI3K/AKT activation not only activates mammalian target of rapamycin (mTOR), but also inhibits Bcl-xL/Bcl-2-associated death promoter (BAD) and BCL2-associated X protein (BAX) expression and phosphorylates MDM2, which causes downregulation of p53-mediated apoptosis and forkhead transcription factors-mediated cell-death [12]. Moreover, PI3K/AKT pathway activation promotes the nuclear factor kappa-light-chain-enhancer of activated B cells (NF-κB), which regulates cancer cell apoptosis, cell cycle, immune modulation, cell survival, cell adhesion and differentiation [12]. Therefore, the upstream PI3K inhibitors may be superior to the downstream mTOR inhibitors in reversing EGFR-TKI resistances. Various of PI3K inhibitors have been compared in SQCLC cells carrying *PIK3CA* mutation, and the superiority of BYL719 to BKM120 or BEZ235 was observed in vitro and in vivo experiments [17]. In KRAS-mutant NSCLC cells, BYL719, combining with selumetinib (a MEK1/2 inhibitor), have exhibited cytotoxicity in vitro and in vivo [29]. Our data demonstrated the efficacy of BYL719 on reversing EGFR-TKI resistance, not only in *PIK3CA* mutated HCC-827-PIK3CA-M and PC-9-PIK3CA-M cells, but also in PI3K/AKT activated cells due to *ErbB2* amplification (H1975) or *PTEN* deletion (H1650). Moreover, the synergistic effect of gefitinib and BYL719 was also confirmed in the 3D cell culture model, the in vivo model, as well as in the organoid model from a lung adenocarcinoma patient with *EGFR*, *ErbB2*, *PIK3CA* mutations and *MET* amplification. It’s worth noting that the patient’s disease has progressed after the treatment for afatinib alone or the combination of afatinib and crizotinib. Our study indicates that the combination of gefitinib and BYL719 may improve the patient’s prognosis, which provides strong evidence for translational medicine. As the first oral PI3K inhibitor selectively targeting the p110α isoform, BYL719 has been approved by FDA for the treatment of breast cancer, indicating the drug safety in human [16]. Therefore, combination of BYL719 and EGFR-TKIs may be a potential effective and safe strategy for *EGFR* mutant NSCLC patients with PI3K pathway aberrations, and warrant further clinical studies.

We also explored the mechanism of the function of the combined effect of gefitinib and BYL719. The results demonstrated that the combination of gefitinib and BYL719 significantly inhibited the phosphorylation of AKT, when compared with control group and gefitinib group in the *EGFR* mutant NSCLC cell lines with PI3K/AKT activation in 2D/3D culture and in PC-9-PIK3CA-M xenografts. AKT, the key effector of PI3K/AKT signaling, is a member of the AGC (PKA/PKG/PKC) protein kinase family and consists of three homologues [12]. AKT activation inhibits the expression of BAD and BAX, and phosphorylates MDM2, which causes downregulation of p53-mediated apoptosis and forkhead transcription factors-mediated cell-death [12]. Moreover, PI3K/AKT pathway activation promotes the nuclear factor kappa-light-chain-enhancer of activated B cells (NF-κB), which regulates cancer cell apoptosis, cell cycle, immune modulation, cell survival, cell adhesion and differentiation [12]. Another important downstream pathway resulting from AKT activation is activation of the protein kinase, mTOR [30]. BKM120 and BYL719 downregulated the members of the PI3K/AKT/mTOR pathway in a dose-dependent manner, as indicated by the reduced phosphorylation levels of AKT and p70S6K in SQCLC cell lines carrying *PIK3CA* mutation [17]. Our results showed that the combination of gefitinib and BYL719 downregulated the phosphorylation levels of AKT in NSCLC cell lines carrying *EGFR* mutations and *PIK3CA* mutations/amplification. The in-depth mechanisms need to be explored in the future.

In summary, we have shown that gain of *PIK3CA* mutation may lead to over-growth and gefitinib resistance in *EGFR* mutated NSCLC cells. Combination of BYL719, a PI3Kα specific inhibitor, is able to overcome the resistance to gefitinib induced by PI3K/AKT activation in *EGFR* mutated NSCLC cell lines both in vitro and in vivo, and may be a new treatment strategy for *EGFR* mutated NSCLC patients with PI3K pathway aberrations.

### Electronic supplementary material

Below is the link to the electronic supplementary material.

Supplementary Material 1: The original bands of western blots

Supplementary Material 2: The gene background of NSCLC cells and PIK3CA expression levels of NSCLC cells. Supplementary Fig. 1 PIK3CA mRNA and protein were over-expressed in H1975 and H1650 cells.
Supplementary Table 1 The characterization of *EGFR*, *ErBb2*, *PTEN* and *PIK3CA* in NSCLC cells.
Supplementary Fig. 1 PIK3CA mRNA and protein were over-expressed in H1975 and H1650 cells

## Data Availability

The datasets used and/or analyzed during the current study are available from the corresponding author on reasonable request.
