# Supplementary figures and images for: BYL719 reverses gefitinib-resistance induced by PI3K/AKT activation in non-small cell lung cancer cells
Source: BMC Cancer. 2023 Aug 8;23:732. doi: 10.1186/s12885-023-11243-0 (PMC10408073; doi:10.1186/s12885-023-11243-0)

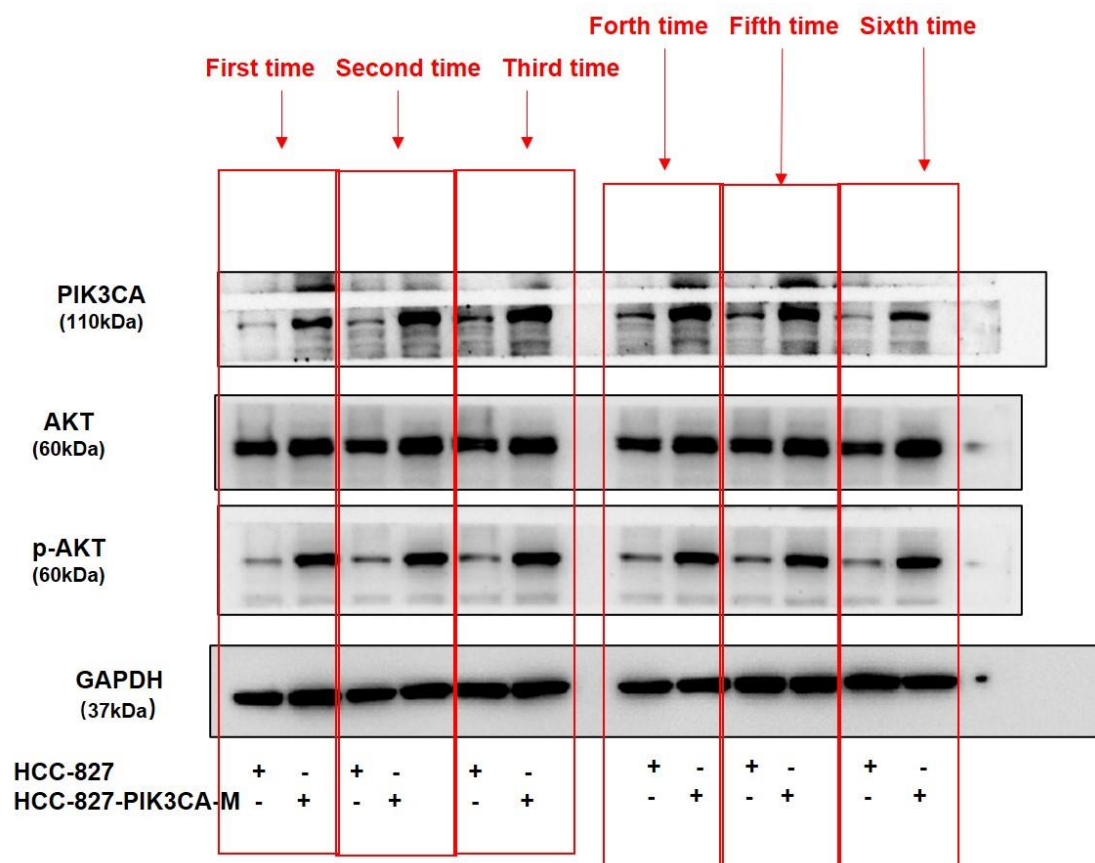

Fig. 1A

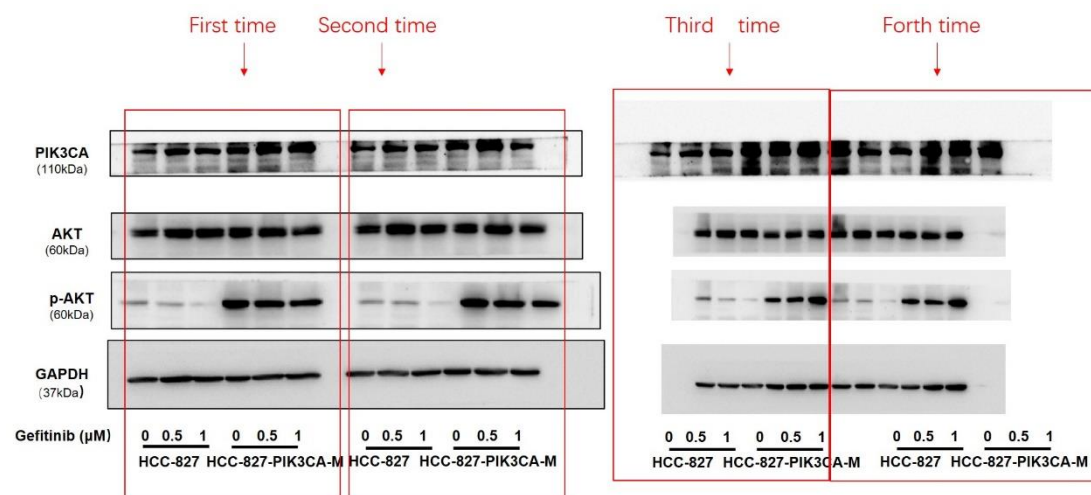

Fig. 1C

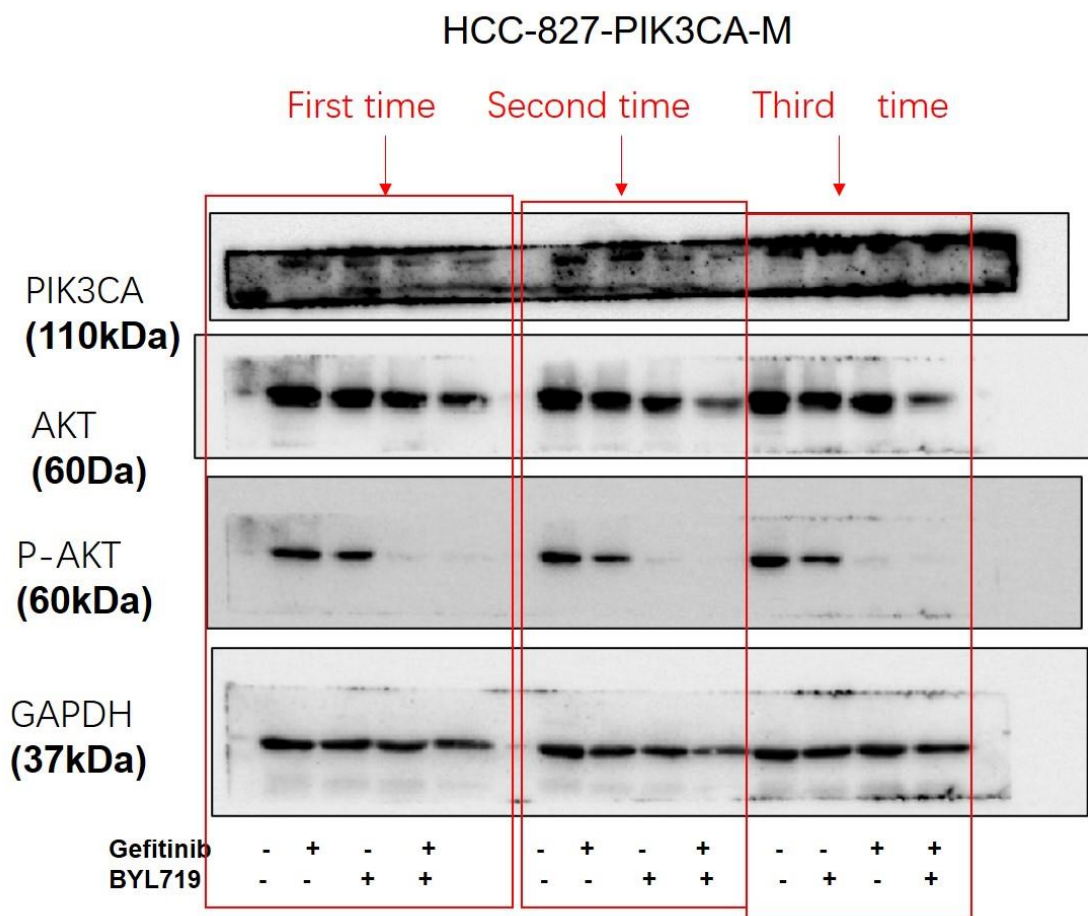

**Fig. 4A**

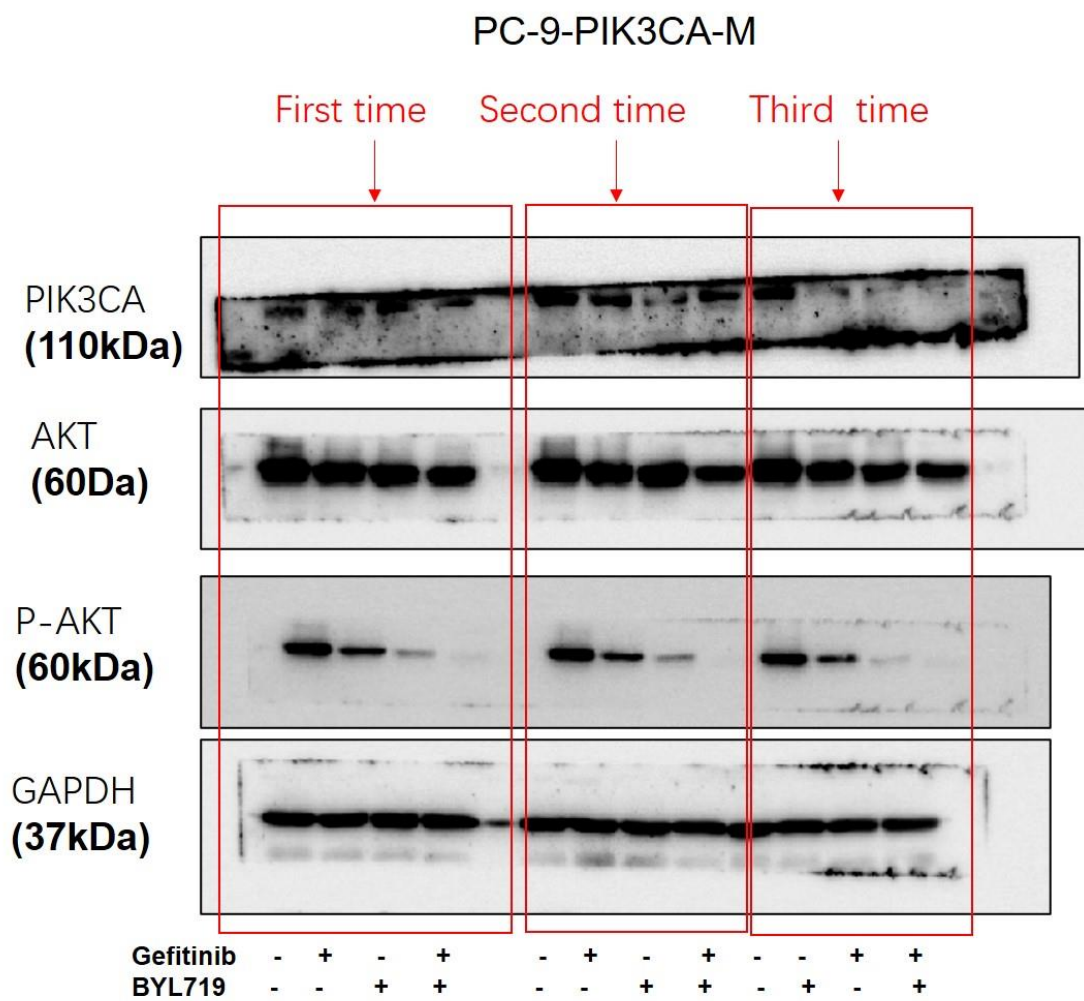

**Fig. 4A**

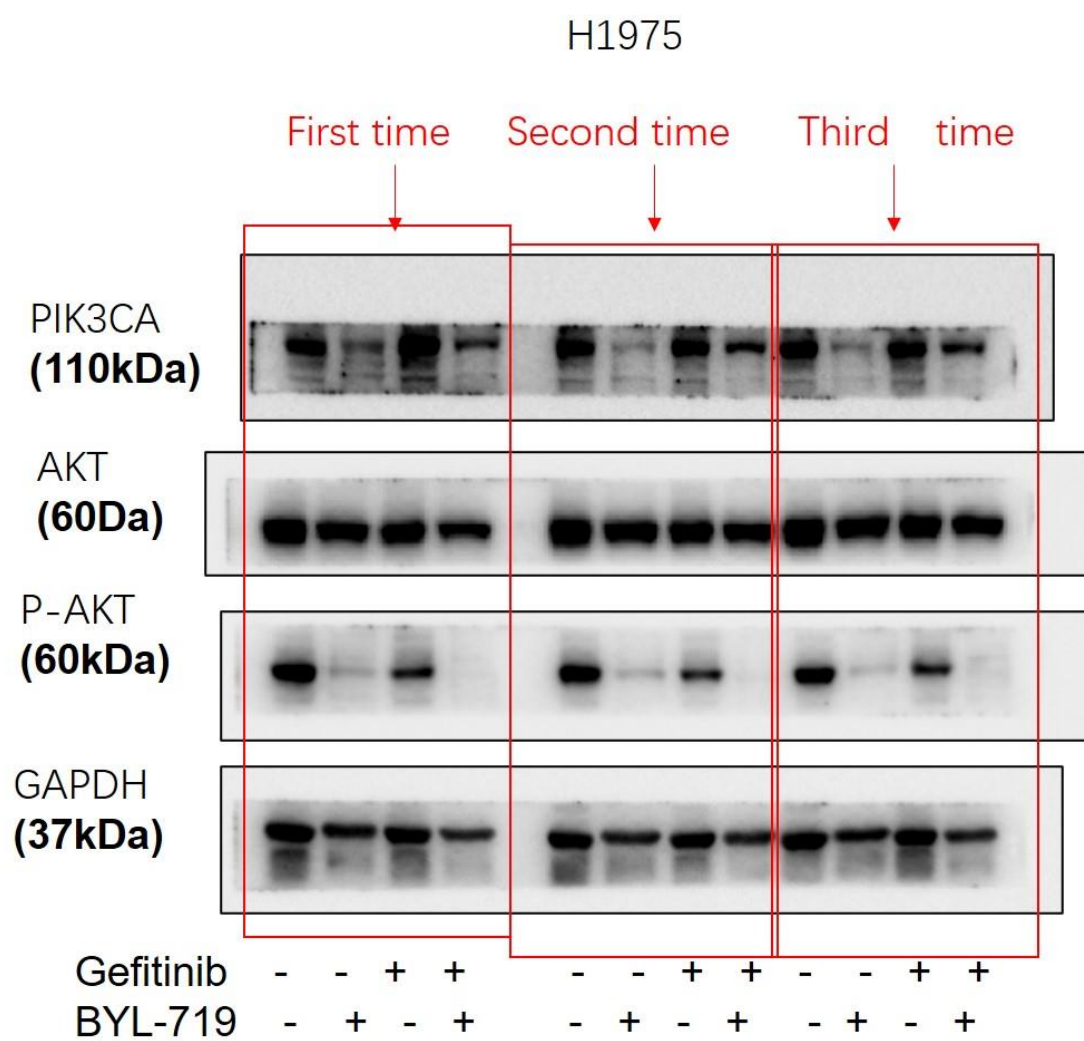

**Fig. 4A**

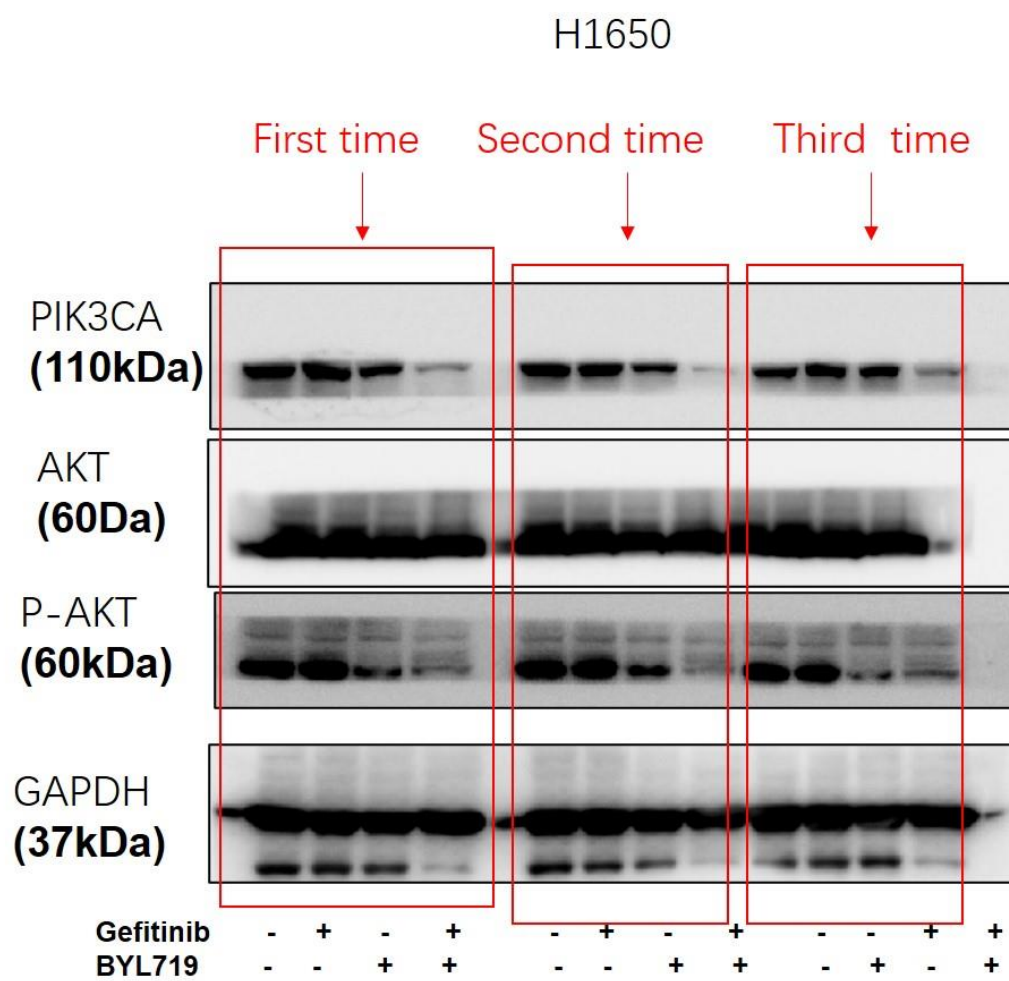

**Fig. 4A**

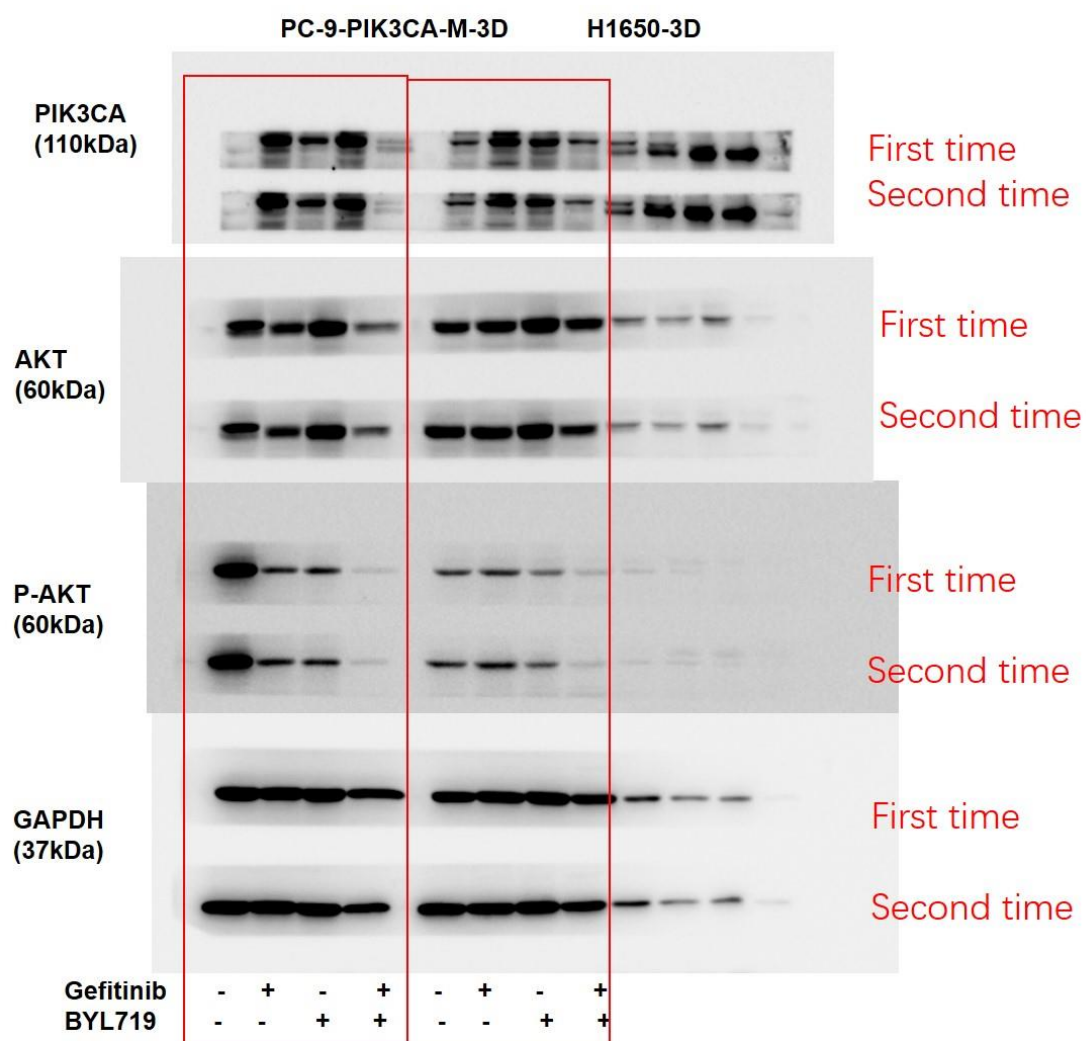

Fig. 5D

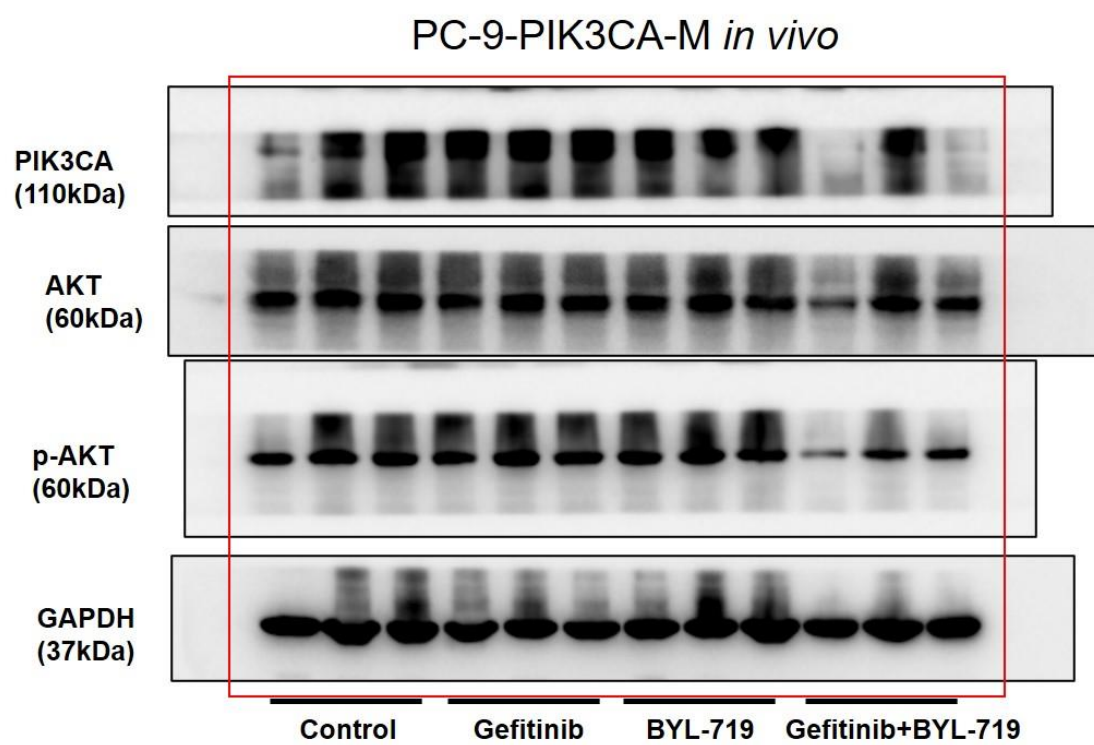

Fig. 6E

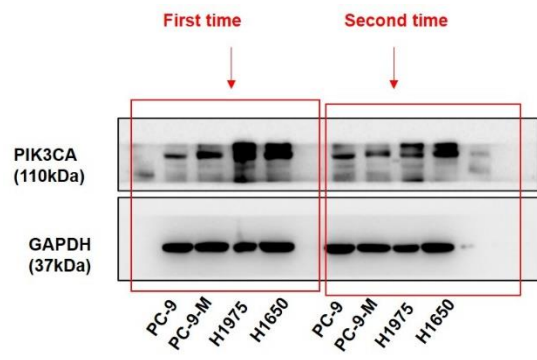

Supplementary Fig. 1B

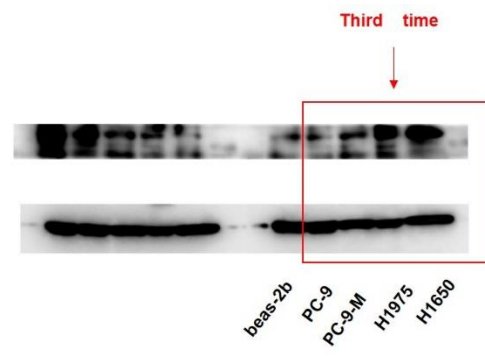

Supplementary Fig. 1B

Supplement: Supplementary file 1 — Supplementary Material 1: The original bands of western blots [file 12885_2023_11243_MOESM1_ESM.pdf]
