# Supplementary material for: BYL719 reverses gefitinib-resistance induced by PI3K/AKT activation in non-small cell lung cancer cells
Source: BMC Cancer. 2023 Aug 8;23:732. doi: 10.1186/s12885-023-11243-0 (PMC10408073; doi:10.1186/s12885-023-11243-0)
Supplement: Supplementary file 2 — Supplementary Material 2: The gene background of NSCLC cells and PIK3CA expression levels of NSCLC cells. Supplementary Fig. 1 PIK3CA mRNA and protein were over-expressed in H1975 and H1650 cells. Supplementary Table 1 The characterization of EGFR, ErBb2, PTEN and PIK3CA in NSCLC cells. Supplementary Fig. 1 PIK3CA mRNA and protein were over-expressed in H1975 and H1650 cells [file 12885_2023_11243_MOESM2_ESM.docx]

**Supplementary Table 1 The characterization of *EGFR*, *ErBb2*, *PTEN* and *PIK3CA* in NSCLC cells**

| Cell lines | *EGFR* | *ErBb2* | *PTEN* | *PIK3CA* |
| --- | --- | --- | --- | --- |
| HCC-827 | Exon19 deletion | WT | + | WT |
| HCC-827-PIK3CA-M | Exon19 deletion | WT | + | Mutant |
| PC-9 | Exon19 deletion | WT | + | WT |
| PC-9-PIK3CA-M | Exon19 deletion | WT | + | Mutant |
| H1975 | L858R mutation | activation | + | WT |
| H1650 | Exon19 deletion | WT | - | WT |

**
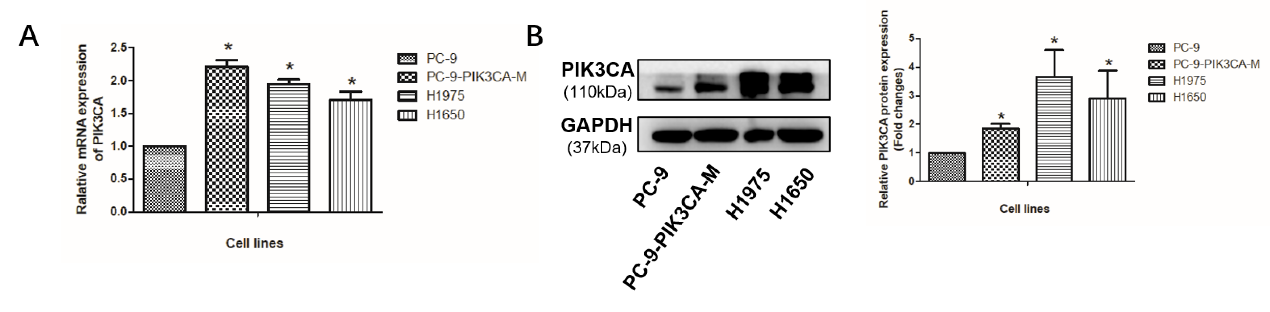
**

**Supplementary Fig. 1 PIK3CA mRNA and protein were over-expressed in H1975 and H1650 cells**

A: The mRNA expression of PIK3CA in PC-9, PC-9-PIK3CA-M, H1975 and H1650 cells; B: The protein expression of PIK3CA in PC-9, PC-9-PIK3CA-M, H1975 and H1650 cells. *P<0.05, PC-9-PIK3CA-M/H1975/H1650 group vs PC-9 group.
